# P-1823. Development of a *Nocardia* Reporter Strain to Study Cell-type Specific Phagocytosis and Killing *in vivo*

**DOI:** 10.1093/ofid/ofae631.1986

**Published:** 2025-01-29

**Authors:** Michael S Abers, Peter M Finin, Li Ma, Adrian Zelazny, Helena Boshoff, Clifton E Barry, Steven M Holland, Michail S Lionakis

**Affiliations:** National Institutes of Health, Bethesda, Maryland; National Institutes of Health, Bethesda, Maryland; National Institutes of Health, Bethesda, Maryland; NIH, Bethesda, MD; National Institutes of Health, Bethesda, Maryland; National Institutes of Health, Bethesda, Maryland; National Institutes of Health, Bethesda, Maryland; National Institute of Allergy and Infectious Diseases (NIAID)/National Institutes of Health (NIH), Bethesda, MD

## Abstract

**Background:**

*Nocardia* infections are associated with substantial morbidity and mortality. Little is known about how the immune system defends against *Nocardia*.

**Methods:**

*Nocardia* was transformed to contain a genomic copy of mScarlet. The cell wall was then labelled with AlexaFluor633 (AF633) to generate dually labelled (mScarlet^+^ AF633^+^) *Nocardia*. *Cybb* knockout (KO) mice (chronic granulomatous disease or CGD) and wildtype (WT) controls were inoculated with 5-10 million colony forming units (CFU) via oropharyngeal aspiration. High-dimensional flow cytometry (HDFC) and bacterial burden were determined in lung and bronchoalveolar lavage (BAL) fluid obtained 24 hours post-infection. Phagocytic index (PI) was defined as % of cells that are AF633^+^ cells. Killing capacity (KI) was defined as % of AF633^+^ cells that are mScarlet^–^.

**Results:**

Live cultures of dually labelled *Nocardia* were positive for both mScarlet and AF633. mScarlet but not AF633 fluorescence decreased to undetectable levels upon killing of *Nocardia*. HDFC demonstrated that alveolar macrophages (AM), interstitial macrophages (IM), neutrophils, monocytes, and type 2 dendritic cells (cDC2) are the primary cell types that interact with *Nocardia in vivo*. PI was highest in AM and IM. PI was greater in cells from BAL versus lung (P< 0.01) while the opposite was true for other cell types. KI was lower in neutrophils versus other cell types (P< 0.05). Mortality and bacterial burden in lung were higher in CGD mice compared to WT controls (P< 0.01 for both comparisons). CGD mice demonstrated reduced PI in neutrophils, AM, IM (P< 0.05 for both). KI was impaired in AM and neutrophils.

**Conclusion:**

Use of a dually labelled *Nocardia* reporter strain demonstrated cell-type specific defects in both phagocytosis and killing of *Nocardia* in CGD mice. This might explain the underlying predisposition to nocardiosis seen in humans with CGD. Ongoing studies aim to elucidate the molecular basis for the susceptibility to nocardiosis in humans and mice with CGD.

**Disclosures:**

**All Authors**: No reported disclosures

